# Segmentation of lung parenchyma in CT images using CNN trained with the clustering algorithm generated dataset

**DOI:** 10.1186/s12938-018-0619-9

**Published:** 2019-01-03

**Authors:** Mingjie Xu, Shouliang Qi, Yong Yue, Yueyang Teng, Lisheng Xu, Yudong Yao, Wei Qian

**Affiliations:** 1 Sino-Dutch Biomedical and Information Engineering School, Northeastern University, No. 195 Chuangxin Avenue, Hunnan District, Shenyang, 110169 China; 2 Key Laboratory of Medical Image Computing of Northeastern University (Ministry of Education), Shenyang, China; 3 Department of Radiology, Shengjing Hospital of China Medical University, No. 36 Sanhao Street, Shenyang, 110004 China; 4 Department of Electrical and Computer Engineering, Stevens Institute of Technology, Hoboken, NJ 07030 USA; 5 College of Engineering, University of Texas at El Paso, 500 W University, El Paso, TX 79902 USA

**Keywords:** Lung parenchyma, CT, Segmentation, Convolutional neural network, Clustering

## Abstract

**Background:**

Lung segmentation constitutes a critical procedure for any clinical-decision supporting system aimed to improve the early diagnosis and treatment of lung diseases. Abnormal lungs mainly include lung parenchyma with commonalities on CT images across subjects, diseases and CT scanners, and lung lesions presenting various appearances. Segmentation of lung parenchyma can help locate and analyze the neighboring lesions, but is not well studied in the framework of machine learning.

**Methods:**

We proposed to segment lung parenchyma using a convolutional neural network (CNN) model. To reduce the workload of manually preparing the dataset for training the CNN, one clustering algorithm based method is proposed firstly. Specifically, after splitting CT slices into image patches, the *k*-means clustering algorithm with two categories is performed twice using the mean and minimum intensity of image patch, respectively. A cross-shaped verification, a volume intersection, a connected component analysis and a patch expansion are followed to generate final dataset. Secondly, we design a CNN architecture consisting of only one convolutional layer with six kernels, followed by one maximum pooling layer and two fully connected layers. Using the generated dataset, a variety of CNN models are trained and optimized, and their performances are evaluated by eightfold cross-validation. A separate validation experiment is further conducted using a dataset of 201 subjects (4.62 billion patches) with lung cancer or chronic obstructive pulmonary disease, scanned by CT or PET/CT. The segmentation results by our method are compared with those yielded by manual segmentation and some available methods.

**Results:**

A total of 121,728 patches are generated to train and validate the CNN models. After the parameter optimization, our CNN model achieves an average F-score of 0.9917 and an area of curve up to 0.9991 for classification of lung parenchyma and non-lung-parenchyma. The obtain model can segment the lung parenchyma accurately for 201 subjects with heterogeneous lung diseases and CT scanners. The overlap ratio between the manual segmentation and the one by our method reaches 0.96.

**Conclusions:**

The results demonstrated that the proposed clustering algorithm based method can generate the training dataset for CNN models. The obtained CNN model can segment lung parenchyma with very satisfactory performance and have the potential to locate and analyze lung lesions.

## Background

In recent years, segmentation has known great successes in various medical images analysis tasks including detection of atherosclerotic plaques [1], pelvic cavity assessment [2, 3], ear image data towards biomechanical researches [4], skin lesions detection [5], etc. This has led to its expansion to lung diseases detection [6, 7] and specifically to lung field extraction [8]. Lung segmentation is an incredibly important component of any clinical-decision support system dedicated to improving the early diagnosis of critical lung diseases such as lung cancer, chronic obstructive pulmonary disease (COPD), etc. [9]. However, it constitutes a very challenging task [10]. Lung segmentation is difficult to achieve due to the fact that lung pathologies present various appearances different from the normal lung tissue [11, 12]. There exist dozens of lung diseases including the ground-glass opacity, consolidation, cavity, tree-in-bud and micro nodules, nodules, pleural effusion, honeycomb, etc., and each of them possesses different shape, texture, and attenuation information at CT images [13].

With the aim of improving the early diagnosis and treatment of lung diseases, numerous studies have been conducted to segment and analyze both normal and abnormal lung from CT images. Generally, according to the study by Mansoor et al. [12], existing methods can be categorized into four classes; and each of them owns specific advantages and disadvantages. The first class is the thresholding-based methods which set a thresholding (or CT number) interval to create binary partitions [14]. These methods are the fastest, but pathological regions are often not included and various morphological operations are required. The second class is referred to as region-based methods and includes the region growing [15], graph cuts [16, 17], random walk [10, 18, 19], etc. This class of methods is fast and works well with more subtle attenuation variations. However, they presence some deficiencies such as over-segmentation problem and may fail if there exist great number of pathological findings in the lung. The third class is shape-based methods and can be further divided into two sub-classes: atlas-based and model-based. As the prior knowledge of lung shape, an atlas or model is aligned to the target images firstly, and then the atlas or model is transformed geometrically to the best segmentation through an optimization procedure [20–23]. These methods work well only for the lungs with mild and moderate abnormalities, but have difficulties while creating representative model and are computationally expensive. The fourth class is neighboring anatomy guided methods which use the spatial information of surrounding organs (e.g., rib, heart, spine, liver, spleen) to constrain the segmentation [11]. Moreover, a new trend has been becoming obvious for the segmentation of lung, i.e., the combination of different methods generate better results [12, 19]. Other surface-based methods are also available [24], and readers are encouraged to refer to the reviews for further details [12, 25, 26].

Recently, the tremendous success of machine learning techniques has attracted the attention of many researchers resulting in the development of numerous successful machine learning-based lung segmentation methods. For instance, Xu et al. [27] extracted 24 three-dimensional texture features including the first-order, second-order, fractal features, and used Bayesian classifier to discriminate five categories. Yao et al. [28] had extracted 25 features from each 16 × 16 image patch and used support vector machine (SVM) to differentiate normal from abnormal lung regions (pulmonary infection and fibrosis). Similarly, the 130 gray-level co-occurrence features extracted from 21 × 21 × 21 pixel VOI and k-nearest neighbor classifier were used to classify lung parenchyma into normal, ground glass, and reticular patterns [29]. Extracted Mobius invariant shape features and statistical texture features and SVM were also employed to detect and quantify tree-in-bud (TIB) opacities from CT images [30]. Song et al. [31] had extracted 176 texture, intensity and gradient features from each image patch and investigated the performance of four approximative classifiers. Thereafter, to the end of overcoming the difficulties encountered in the processes of features design and selection, some deep learning (i.e., convolutional neural network, CNN) and representation learning methods have been used to address the lung CT images analysis [32, 33].

Nowadays, many machine learning-based algorithms are being used to detect or distinguish various lung abnormalities and the obtained results are combined with the normal lung parenchyma segmented by other traditional methods to detect the complete lung area. Although, these methods can produce satisfactory results, their implementations comprise many processes which may need longer computational time. Moreover, in some machine learning methods especially the deep learning methods, huge amount of image patches need to be labeled or annotated manually; which is a time-consuming process and constitutes a tedious task for the radiologists. Thus, the development of a machine learning-based framework for precise segmentation of lung parenchyma from thoracic CT images will be of great help in analyzing and treating lung diseases. Additionally, an easier and low-cost accurate way for generating massive dataset used in the processes of training and validation of deep learning model is highly needed.

Motivated by the aforementioned, we propose one different strategy to segment lung parenchyma excluding lesions from CT images using a CNN trained with the clustering algorithm generated dataset. This idea originates from the observation that the normal lung parenchyma owns commonalities across subjects, diseases and CT scanners, although lung pathologies present various appearances at CT images. Segmentation of lung parenchyma can help detect and locate neighboring lung lesions, which is of great significance to the early diagnosis and treatment of lung diseases. The contributions of this paper are as follows. First, we proposed a weak supervised approach to generate large amount of CT image patches for the subsequent training and validation of CNN which can be effectively and efficiently used to replace the conventional time-consuming process of determining regions of interest (ROI) manually. Second, we designed and trained a CNN model to identify patches of lung parenchyma generated from CT images. Through this trained CNN model, the fully automatic segmentation of lung parenchyma can be achieved with excellent robustness, efficiency and accuracy. The proposed fully automated machine learning based framework for lung parenchyma segmentation possesses the potential to help researchers and radiologists locate and analyze the neighboring lesions of the lung.

## Methods

Our proposed lung parenchyma segmentation method consists of three stages: (1) the generation of the labeled dataset to be fed into the CNN; (2) the design, training and validation of a CNN model; (3) the segmentation using the trained CNN. A detailed explanation of every stage of the proposed framework is given below.

### The generation of the labeled dataset

Adopting the popular machine learning framework, all the input images with a fixed size of 512 × 512 are split into smaller patches with the same size at first. The size of patches is determined through comparing the clustering results at different settings of 64 × 64, 32 × 32, 16 × 16, 8 × 8, 4 × 4 and 2 × 2. The best patch size is set to 8 × 8, and the reason will be interpreted in the subsection of experiments. Total number of patches is 10,076,160 split from the data of 23 patients.

**Fig. 1:**
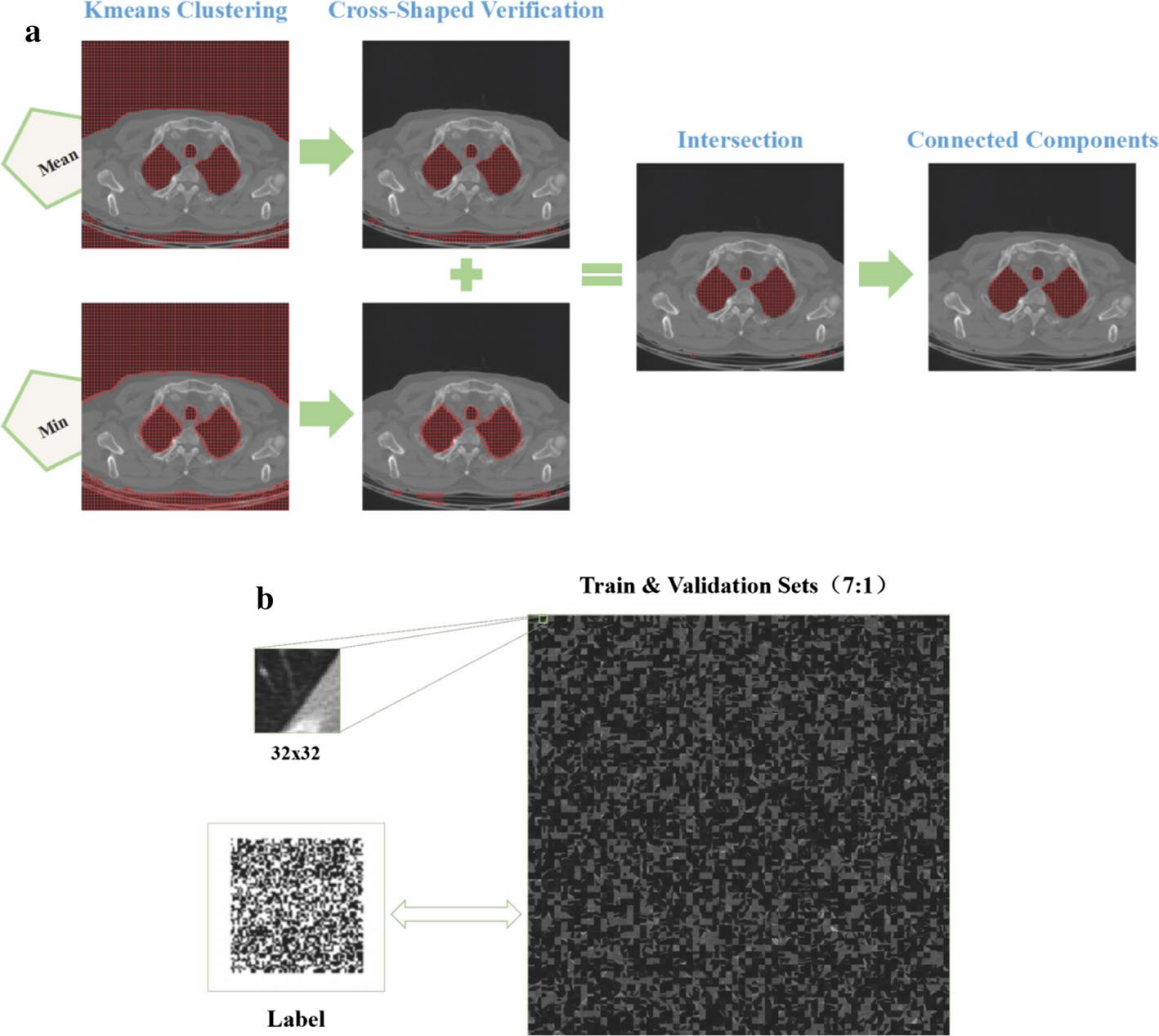
The generation of the labeled data. **a** The procedures and corresponding results after each sub-step. **b** The generated dataset for the training and validation of the CNN

The complete procedure with corresponding results after each sub-step of generating the labeled dataset is illustrated in Fig. 1. At first, with all the 10,076,160 patches as input, the *k*-means clustering algorithm with two categories is performed twice using the mean and minimum intensity of the image patch, respectively. As shown in Fig. 1a, the lung parenchyma and the background air outside the human body have been grouped into one class of low intensity. Then, one technique named the cross-shaped verification is used to remove the group of patches of that background air. For each group of low intensity patch obtained in this step, we check whether there is at least a high intensity patch in all of its four directions (left, right, up, and down). Only if this assumption is true, the current low intensity patch will be kept and regarded as one of lung parenchyma, otherwise it will be discarded. The *k*-means clustering with mean intensity of patch and the cross-shaped verification possesses the ability to generate more accurate lung parenchyma boundaries, but more patches are kept in the gap between human body and the scanner bed. The same process occurs in the opposite way in the case of using the minimum intensity. Hence we apply this kind of *k*-means clustering algorithm twice and take the intersection of the obtained volumes.

Subsequently, connected component analysis algorithm based on Max-Tree proposed by Fu et al. [34] is applied to extract the lung parenchyma. Furthermore, padding is performed to expand the 8 × 8 patch into 32 × 32 patch without overlapping so as to meet the image input demand of the next CNN training, as shown in Fig. 1b. It is worth mentioning that the expansion of the 8 × 8 patch will result in image patch containing both lung parenchyma and body parts. However, the center of every patch is the lung parenchyma. Finally a total of 60,864 patches of lung parenchyma are generated. Correspondingly, the same number of patches belonging to non-lung parenchyma is selected out randomly for the balance of two classes. The balance of the two classification classes is performed in the aim of eliminating the decline in testing accuracy caused by the imbalance of the training dataset.

### A CNN model

**Fig. 2:**
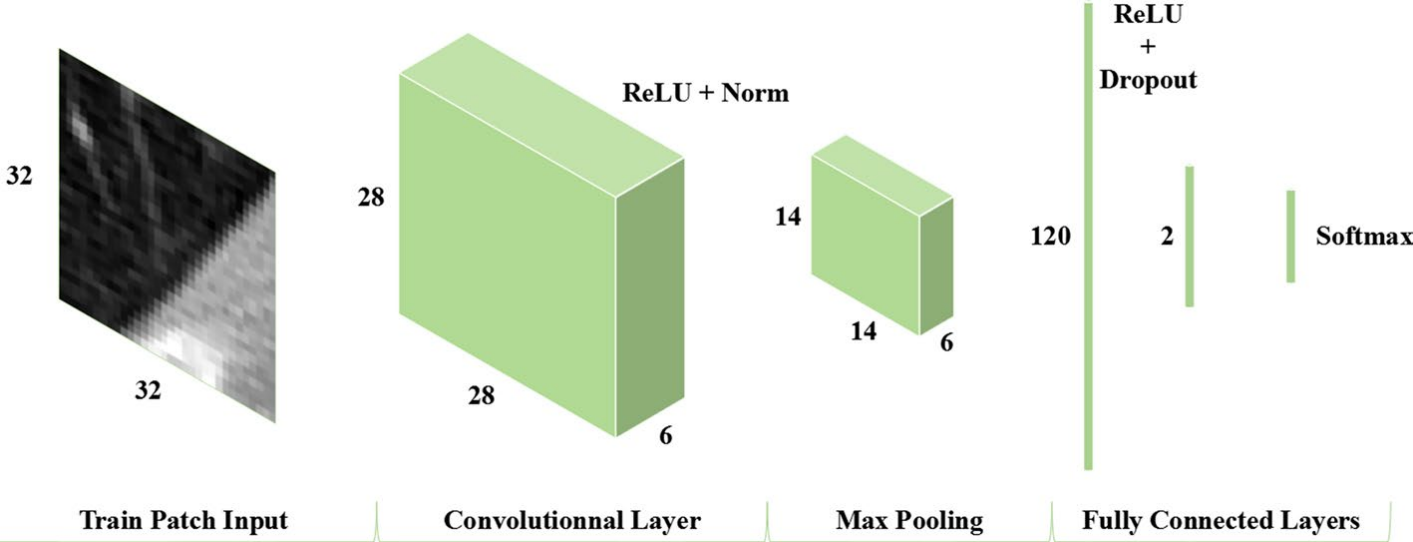
The network architecture of the proposed simplified CNN

We proposed a simplified CNN model possessing the ability to differentiate the real lung parenchyma image patches from non-lung parenchyma image patches. The structure of this CNN network comprises an image input layer, a convolutional layer, a pooling layer and two fully connected layers with a Softmax layer. A detailed description of the abovementioned CNN model is displayed in Fig. 2. Comparing with the well-known AlexNet structure comprising five convolutional layers, we just preserve one convolutional layer with six convolutional kernels to deal with the input set of image patches of 32 × 32. The single convolution layer is followed by rectified Linear Unit (ReLU) and normalization layers which helps accelerate the convergence of the stochastic gradient descent (SGD) and prevent overfitting as well. Then a MaxPooling layer, the first fully connected layer, a dropout layer, ReLU layer, the second fully connected layer and Softmax layer follow, respectively. The first fully connected (FC) layer includes 120 neurons. The dropout layer helps in avoiding overfitting.

### The segmentation using CNN

After splitting all the CT images for segmentation into patches of 32 × 32 using each voxel as the center point, they are input into the trained CNN. Simultaneously, each patch will be automatically given a label of 1 or 0, denoting lung parenchyma (LP) or non-lung parenchyma (NLP). The maximum connected component detection is done to extract the whole LP volume. Finally the hole in the LP volume is filled to achieve the final segmentation results of lung parenchyma.

## Experiments and image data

In the aim of implementing the machine learning-based framework proposed in this study, four main experiments have been conducted. The first experiment consists of determining the most appropriate patch size among different sizes including 64 × 64, 32 × 32, 16 × 16, 8 × 8, 4 × 4 and 2 × 2; during the stage of generating the labeled dataset of LP and NLP. The characteristics of the image patches and the computational time are assessed to determine the best patch size. The second experiment is the parameters optimization procedure of the CNN model; which will be described shortly in “CNN parameters optimization” section. The performance of the trained CNN model is tested in the third experiment. The experimental dataset used in the second and third experiments consists of 121,728 patches obtained from “The generation of the labeled dataset” section. The fourth experiment is performed using a separate dataset of 201 subjects diagnosed with lung cancer or chronic obstructive pulmonary disease (COPD) to further evaluate the performance of the trained CNN model. The details of the partition of the experimental dataset into training, validation and testing are given in Table 1.

**Table 1:** The details of the train/validation dataset and the separate dataset

Dataset	Number of patients	Number of slices	Number of lung parenchyma patches	Number of non-lung parenchyma patches	Total number of patches
The train/validation	23	2460	60,864	60,864	121,728
The separate	201	19,967	415,612,531	4.2040 × 10^9^	4.6196 × 10^9^

All the experiments of this study were conducted under a Windows 7 on a workstation with CPU Intel Xeon E5-2620 v3 @2.40 GHz, GPU NVIDIA Quadro K2200 and 32 GB of RAM. The proposed CNN was implemented using the simplified AlexNet structure and the procedures of unsupervised clustering generation algorithm were implemented in MATLAB 2017a.

### CNN parameters optimization

To the end of setting the best values of the CNN parameters, numerous experiments were conducted while evaluating its performance through comparing the achieved average value of *F*-score (*F*_*avg*_) and the computational time of training of the training process. *F*_*avg*_ is defined as

1 $$F_{\text{avg}} = = \frac{{Precision_{nlp} \times Recall_{nlp} }}{{Precision_{nlp} + Recall_{nlp} }} + \frac{{Precision_{lp} \times Recall_{lp} }}{{Precision_{lp} + Recall_{lp} }}$$

where *Precision*_*nlp*_ and *Recall*_*nlp*_ represent the positive prediction rate and the sensitivity of the class of non-lung parenchyma, respectively. Similarly, *Precision*_*lp*_ and *Recall*_*lp*_ are he positive prediction rate and the sensitivity of the class of lung parenchyma, respectively. *Precision* and *Recall* can be computed as

2 $$Precision = \frac{TP}{TP + FP}$$

3 $$Recall = \frac{TP}{TP + FN}$$

where *TP* is true positive, *FP* is false positive, and *FN* is false negative.

We varied up to 23 parameters during the training process of our CNN model, however, only the variation of nine of those parameters had a significant effect on the classification results. The default settings of these nine parameters can be determined as: the kernel size (5); the kernel number (6); the local response normalization layer (3); the output size of fully connected layer (120); the dropout probability (0.5); the pooling type (Max); the batch size (128); the number of epochs (50); the learning rate (0.01). Using these default settings as the reference, we adjusted each parameter while keeping the others constant and investigated the variation of *F*_*avg*_ and the elapsed time. Specifically, 11 cases were evaluated under the circumstances of the kernel size of 10, the kernel number of 3, the channels of normalization of 1, the output of FC of 240, the dropout probability of 0.2 and 0.1, the pooling type of Avg, the batch size of 256, the epochs of 80, the learning rate of 1 × 10^−5^ and 1 × 10^−4^.

### Performance evaluation using cross-validation

A total of 121.728 image patches of 32 × 32 are divided into the training and validation datasets with a ratio of 7:1, and the 8-folder cross-validation is carried out. The relationship between the training accuracy and loss and the number of iterations is investigated. The receiver operating characteristic (ROC) curve is drawn and the area under the ROC curve (AUC) is calculated for the trained CNN model. The confusion matrix and six convolutional kernels are presented at last.

### Performance evaluation using the separate dataset and manual segmentations

One separate dataset containing 201 cases of patients was collected to evaluate the robustness, efficiency and accuracy of the trained CNN model for lung parenchyma segmentation. Among them, nine cases are patients with COPD confirmed by the pulmonary function test, and 192 cases are with lung cancer confirmed by the histopathology examination. For the cases with lung cancer, 174 cases are acquired by CT scanner, 18 cases by PET/CT scanner, whose CT images have a circular field of view. The 19,967 image slices resulted from examining all the 201 patients’ image files have been split into 4.62 × 10^9^ image patches. The robustness is evaluated through the 201 cases data with different diseases (COPD or lung cancer) and acquired by different scanners (CT and PET/CT). The accuracy is calculated through comparing the lung field segmentation results achieved by the automated CNN model with that yielded by the manual and independent annotations of two experienced radiologists as the reference.

For a more comprehensive and clearer performance evaluation of the proposed machine learning based lung parenchyma segmentation method, four evaluation metrics have been considered including: the Dice similarity coefficient (*DSC*), Hausdorff distance, sensitivity, and specificity.

*DSC* is defined as

4 $$DSC\left( {V_{\text{GT}} ,V_{\text{test}} } \right) = 2\frac{{\left| {V_{\text{GT}} \bigcap {V_{\text{test}} } } \right|}}{{\left| {V_{\text{GT}} } \right| + \left| {V_{\text{test}} } \right|}}$$

where *V*_*GT*_ is the reference standard segmentation (ground truth) obtained by the radiologists manually, *V*_*test*_ is the segmentation by our proposed method.

Haussdorf distance (HD) is to measure how far apart the boundaries of our segmentation and reference (ground truth) are from each other. Let the real lung boundaries (ground truth) obtained by the radiologists and the segmentation by our proposed method be defined by *V*_*GT*_ and *V*_*test*_, respectively. *V*_*GT*_ comprises a set of points $V_{GT(i)} (i = 1,2 \ldots \ldots n)$ and *V*_*test*_ as well comprises a set of points $V_{test(j)} (j = 1,2 \ldots \ldots n)$ [35]. Hence, HD is defined as

5 $$HD\left( {V_{GT} ,V_{test} } \right) = \hbox{max} \left( {\mathop {\hbox{max} }\limits_{{i \in V_{GT} }} \mathop {\hbox{min} }\limits_{{j \in V_{test} }} \left\| {i - j} \right\|,\mathop {\hbox{max} }\limits_{{j \in V_{test} }} \mathop {\hbox{min} }\limits_{{i \in V_{GT} }} \left\| {i - j} \right\|} \right)$$

Sensitivity is defined as *TP*/*P*, where *P* is the number of voxels in reference and *TP* is the number of voxels segmented correctly by the proposed method. Specificity is defined as *TN*/*N*, where *N* is the number of voxels not in reference, *TN* is the number of voxels correctly identified as non-lung parenchyma by the current method.

## Results

### The patch size

**Fig. 3:**
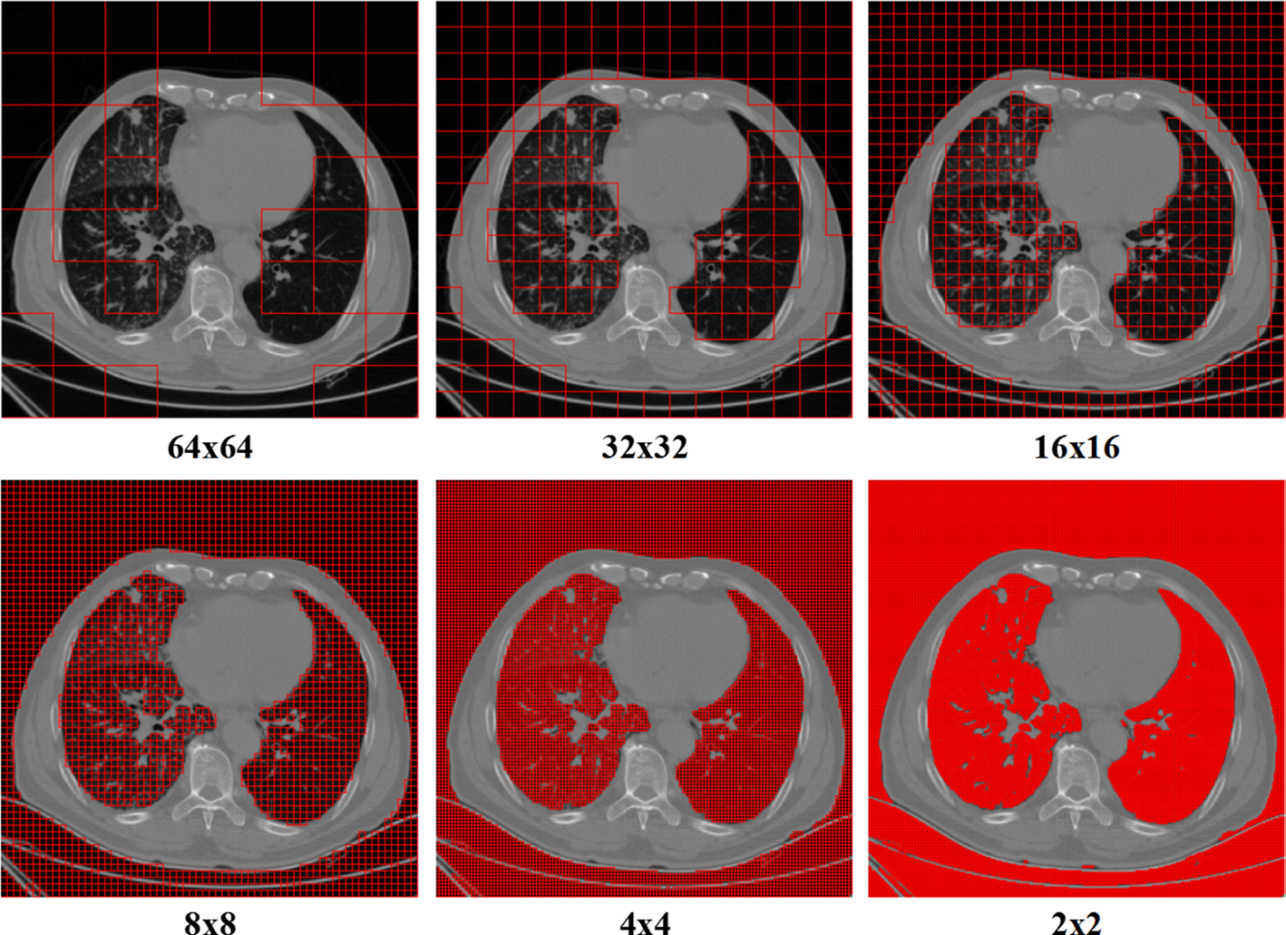
Segmentations using *k*-means clustering with different patch sizes

For different sizes of the image patch, the computational time, characteristics of segmentation, and the assessment are given in Table 2 and Fig. 3. The segmentation results obtained considering six different patch sizes after *k*-means clustering with the mean intensity of the patches for one subject with 126 slices are illustrated in Fig. 3. It can be found that the segmentation becomes more exquisite with the decrease of the patch size, while the computational time increases. Moreover, the accepted results can be obtained while the size is equivalent to or smaller than 8 × 8. The nodule and regions with high CT number are excluded from the lung parenchyma. Considering the tradeoff between the time-computation cost and the achieved segmentation results, the patch size of 8 × 8 is set as the most appropriate choice.

**Table 2:** The assessment of patch size through the segmentation characteristics and time consumption

Patch Size	Time consumption for one patient	Characteristics of patch segmentation	Assessment
64 × 64	0.9306 s	Too rough, fast	Not considered
32 × 32	1.2909 s	Rough, fast	Bad choice
16 × 16	2.6340 s	Including other tissues such as fat, tumor and heart	Bad choice
4 × 4	25.8358 s	Exquisite, but computationally expensive	Second choice
2 × 2	98.0321 s	Very exquisite, but computationally expensive	Bad choice
8 × 8	7.5695 s	Exquisite	Best choice

### Optimization of the CNN parameters

Experiments were conducted considering the default settings of the nine parameters previously mentioned in “CNN parameters optimization” section whose numerical values are shown in the first row of Table 3. The proposed system yielded a *F*_*avg*_ validation of 0.9758 and the elapsed time for completing the training and validation was 846.53 s. Using these settings as the reference, each parameter is adjusted while keeping the others constant and the variation of *F*_*avg*_ and the elapsed time are evaluated. The optimization of the network parameters through performing experiments while varying their values was conducted in the following manner. Doubling the size of the convolutional kernel from 5 × 5 to 10 × 10 led to the drop of *F*_*avg*_ from 0.9758 to 0.9688 with an increase of the elapsed time by 27%. *F*_*avg*_ also decreases slightly for both cases of the kernel number of 3 and the local response normalization layer of 1. Doubling the output size of the fully connected layer from 120 to 240 led to a slight increase of *F*_*avg*_ from 0.9758 to 0.9765, meanwhile the network training time rises sharply to 1282.9 s. The dropout probability of 0.5 has been chosen to be the optimal because the *F*_*avg*_ reached 0.9688 and 0.9541 for a dropout probability of 0.2 and 0.1 respectively. In opposition to its usual effects on the training results, an increase of the batch size from 128 to 256 resulted in a diminishment of the *F*_*avg*_ from 0.9758 to 0.9659. Similarly, an augmentation of the epoch’s number to 80 resulted in a decrease of the *F*_*avg*_ value. Last but not least, the learning rate is a very critical parameter whose decrease can significantly increase the *F*_*avg*_ value. Specifically, the *F*_*avg*_ reaches 0.9855 and 0.9917 for the learning rate of 0.00001 and 0.0001, respectively. The final optimized parameters employed in our trained CNN model can be found in the last row of Table 3. In summary, the relationships between *F*_*avg*_ and most training parameters are not monotonic, and there exists an optimum condition for exploration.

**Table 3:** Performance of the proposed CNNs with different parameters

Kernel size	Kernel number	Channels of normalization	Output of FC	Dropout probability	Pooling type	Batch size	Epochs	Learning rate	Validation *F*_avg_	Elapsed time
*5*	*6*	*3*	*120*	*0.5*	*Max*	*128*	*50*	*0.01*	*0.9758*	*846.53*
*10*	6	3	120	0.5	Max	128	50	0.01	0.9688	1077.55
5	*3*	3	120	0.5	Max	128	50	0.01	0.9609	806.078
5	6	*1*	120	0.5	Max	128	50	0.01	0.9657	997.13
5	6	3	*240*	0.5	Max	128	50	0.01	0.9765	1282.90
5	6	3	120	*0.2*	Max	128	50	0.01	0.9688	1593.91
5	6	3	120	*0.1*	Max	128	50	0.01	0.9541	1589.37
5	6	3	120	0.5	*Avg*	128	50	0.01	0.9682	1606.79
5	6	3	120	0.5	Max	*256*	50	0.01	0.9659	905.27
5	6	3	120	0.5	Max	128	*80*	0.01	0.9722	2660.79
5	6	3	120	0.5	Max	128	50	*0.00001*	0.9855	1566.26
*5*	*6*	*3*	*120*	*0.5*	*Max*	*128*	*50*	*0.0001*	*0.9917*	*1609.34*

The kernel size and the kernel number are only related to the convolutional layer. FC represents the first fully connected layer in our proposed CNNs. The elapsed time indicates the time for training the CNN in different epochs

The Italic in the first row indicates the default setting of parameters. The Italic in final row indicates the final optimized setting of parameters. The Italic in other rows indicates the modifed parameter compared with the default setting

### Performance evaluated using cross-validation

**Fig. 4:**
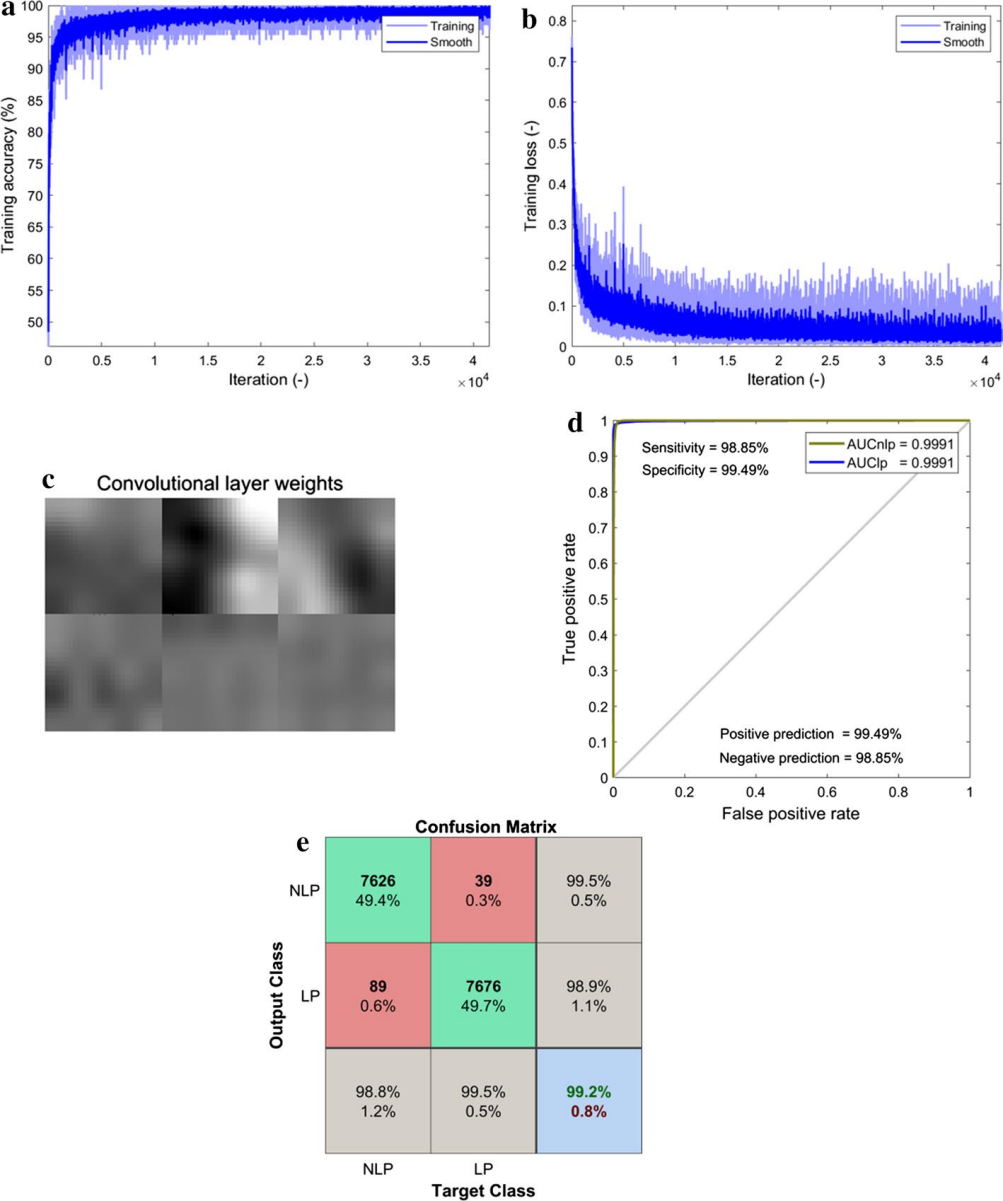
Performance of the trained CNN for lung parenchyma segmentation. **a** The training accuracy. **b** The training loss. **c** The six convolutional kernels. **d** The receiver operating characteristic (ROC) curve. **e** The confusion matrix

Using the hyper-parameter settings recorded in the last row of Table 3, the training accuracy and loss as the function of iterations number are presented in Fig. 4a, b respectively. The training accuracy and loss reach 99.08% and 0.0294, respectively, while the number of iteration reaches to 4.15 × 10^4^, indicating the convergence without overfitting during the training process of our designed CNN model. The visualization of the six kernels of the convolutional layer after the lung parenchyma segmentation process is displayed in Fig. 4c. It is easily noticeable that the image patterns are very smooth and there is absence of noises and artifacts. Thus, the network parameters have been appropriately chosen leading to great classification performance. In addition, the ROC curve of the trained CNN is plotted in Fig. 4d represents, which clearly displays that the area under the curve (AUC) is up to 0.9991 for both lung parenchyma and non-lung parenchyma categories. Meanwhile, the confusion matrix shown in Fig. 4e indicates a sensitivity of 98.8%, a positive prediction of 99.5%, a specificity of 99.5%, a negative prediction of 98.9%, and an accuracy of 99.2%.

### Performance evaluated using the separate dataset and manual segmentations

**Fig. 5:**
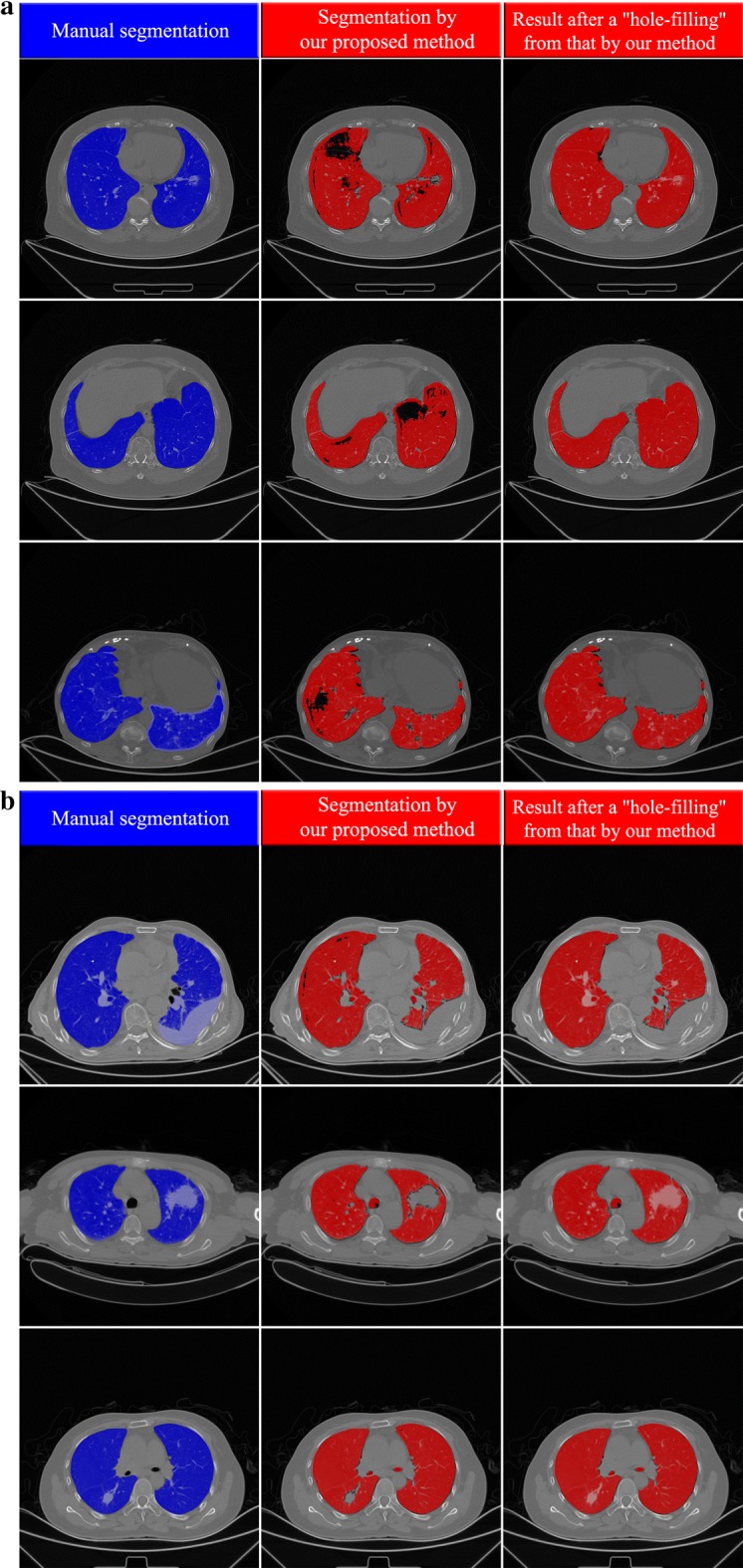
Examples of segmentation at axial slices (In each sub-figure, there are three columns: the left the left column shows the results of manual segmentation of lung field including both lung parenchyma and lesions; the middle column presents the results of segmentation of lung parenchyma by our proposed method; the right column shows the results after a “hole-filling” operation from those by our method.). **a** Three example slices (three rows) for subjects with COPD. **b** Three example slices for subjects with lung cancer (CT scanner)

**Fig. 6:**
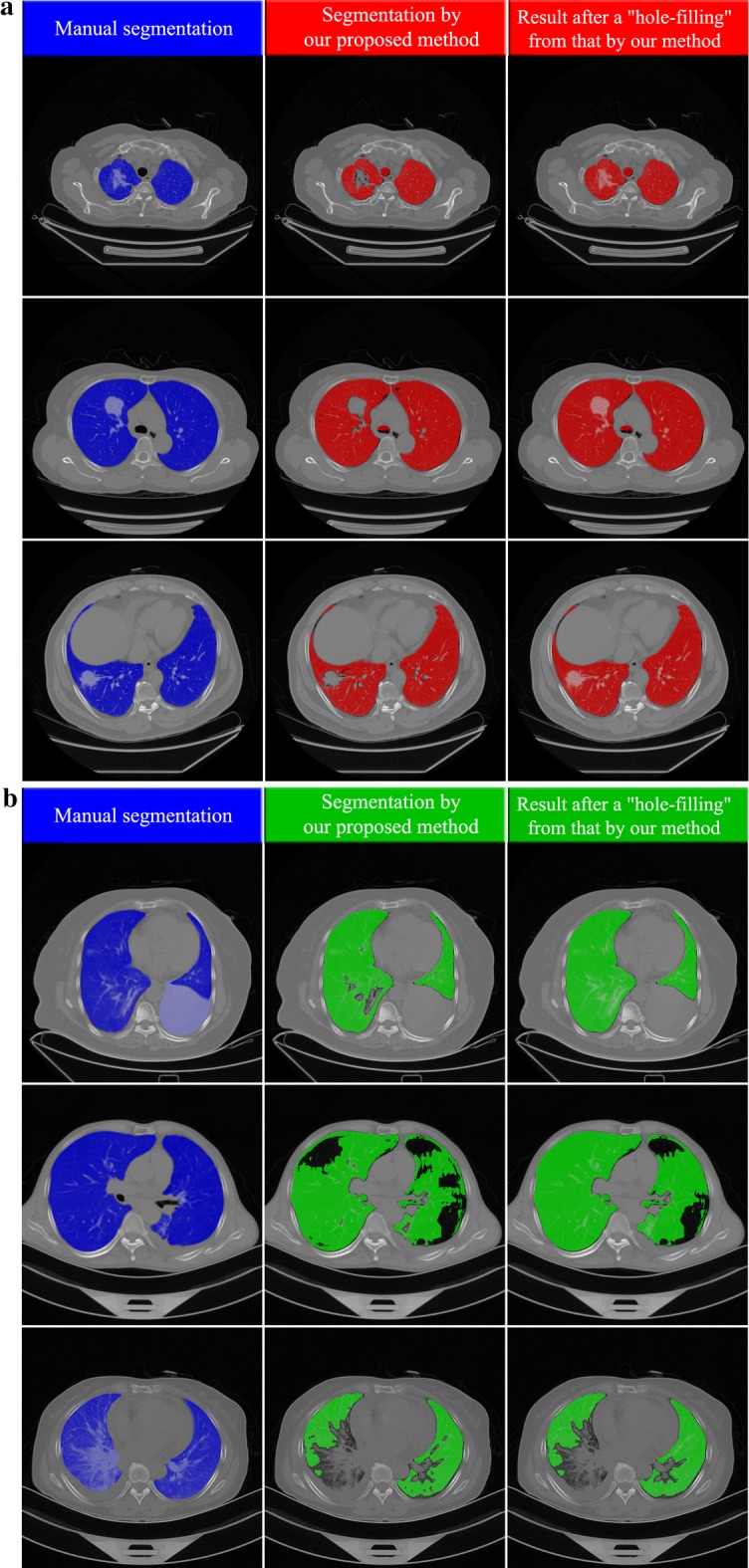
Examples of segmentation at axial slices (In each sub-figure, there are three columns: the left the left column shows the results of manual segmentation of lung field including both lung parenchyma and lesions; the middle column presents the results of segmentation of lung parenchyma by our proposed method; the right column shows the results after a “hole-filling” operation from those by our method.). **a** Three example slices (three rows) for subjects with lung cancer (PET-CT scanner). **b** Three example slices (three rows) for special cases where the pleural effusion, emphysema, inflammation are near the boundary the lung field

Comparing the segmentation results achieved by CNN model with those yielded by the two radiologists for all the 402 slices (manual segmentations), the averaged *DSC*, HD_*avg*_, sensitivity, specificity reach 0.968/0.966, 1.40/1.48 mm, 0.909/0.906, and 0.999/0.999 (to radiologist I/to radiologist II), respectively. For each slice with 512 × 512 voxels, the averaged computational time to segment the region of lung parenchyma is 10.75 s. For a visual illustration of these performances, Figs. 5, 6 displays the segmentation results achieved by our proposed CNN model and manual segmentation on a separate dataset. From left to right, the first, second and third columns represent the segmentation by the radiologists, the segmentation by our CNN model and the hole-filling result performed on our CNN model result, respectively.

Figure 5a presents three segmentation instances of patients suffering from COPD at axial slices. It is found that most of the lung parenchyma regions have been identified and segmented with satisfactory performance. In the second column of Fig. 5a which represents the result of our CNN model, some patches of pulmonary bulla are not well segmented. After an ordinary hole-filling operation, the complete lung field can be obtained, as shown in the third column of Fig. 5a. Besides, there are other three segmentation results of subjects with lung cancer shown in Fig. 5b. Their lung parenchyma can be well distinguished from lung tumor, pleural effusion and other backgrounds. The tumor region embedded in the lung field can be extracted easily through comparing the results before and after hole-filling. Additionally, Fig. 6a demonstrates three more segmentation cases of subjects with lung cancer where the CT images are acquired by PET/CT scanners. One can find that the present CNN model also produces a good effect on lung parenchyma segmentation though the contrast and lung attenuation coefficient are quite different between images acquired by CT and PET/CT scanners.

**Fig. 7:**
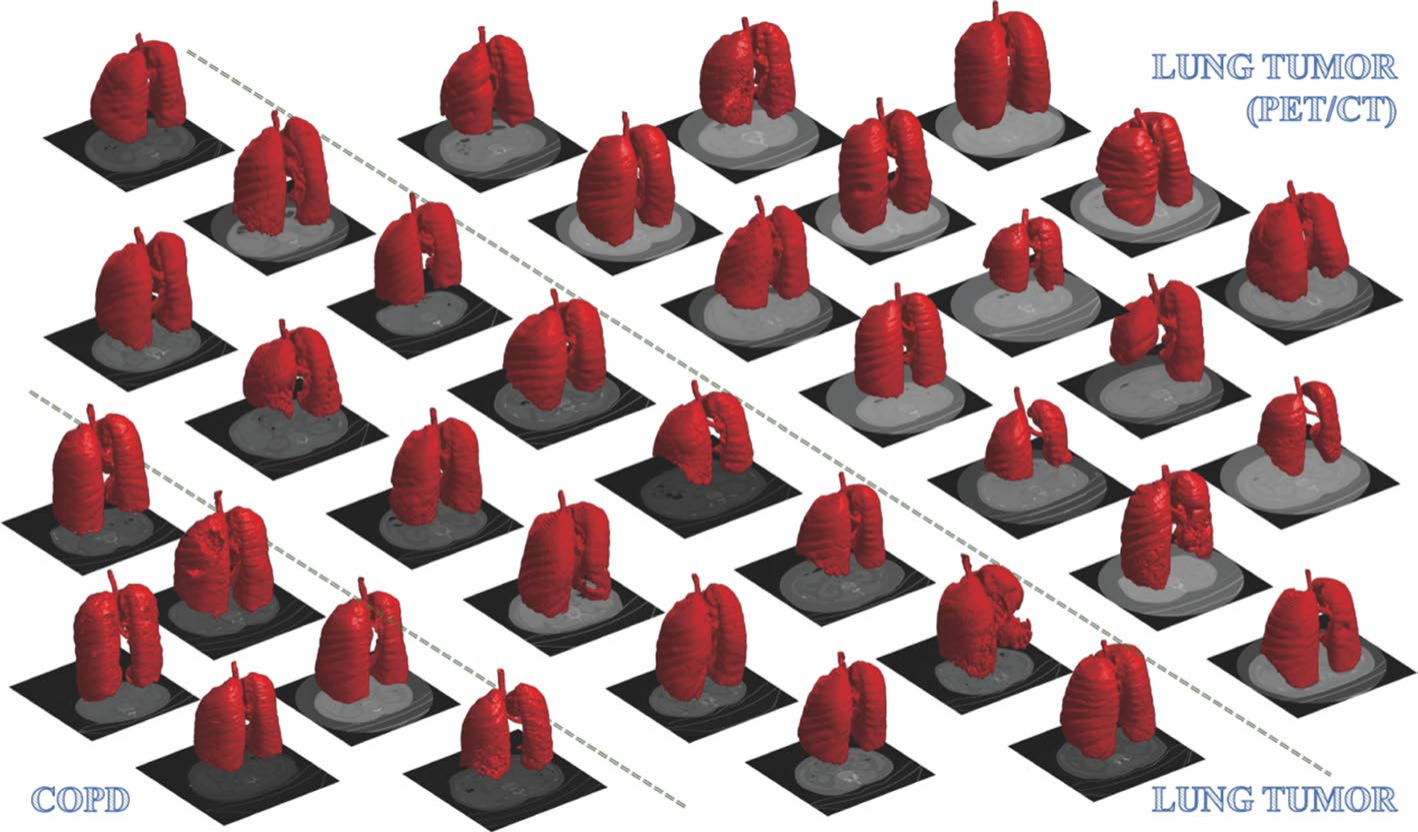
Examples of segmentation shown using 3D surface rendering

Some details on those special cases are shown in Fig. 6b. In the first row, the pleural effusion at the right lung cannot be segmented. The hole-filling result is not able to include these regions, for they are located at the boundary of the lung field. Due to the same reason, the pulmonary bulla near the boundary of lung field also failed to be segmented, as shown in the second row of Fig. 6b. As illustrated in the third row of Fig. 6b, the inflammatory lung cancer cannot be segmented out because its CT features are completely different from those of lung parenchyma. Furthermore, a 3D visualization of some segmentation results is displayed in Fig. 7. For most cases, the segmented lung surface is smooth. There can also be seen some cavities or uncompleted lung fields resulted from the presence of the pleural effusion, pulmonary bulla and lung tumor near the boundary of the lung field.

In sum, from the averaged *DSC* (0.968/0.966), HD_*avg*_ (1.40/1.48 mm), sensitivity (0.909/0.906), specificity (0.999/0.999) yielded by our proposed CNN model and the visualization of the segmentation results shown in Figs. 5, 6 and 7, the proposed deep learning based approach achieved very satisfactory segmentation performance.

## Discussions

### Semi-supervised method of generating annotations

One semi-supervised method of generating annotations has been proposed and implemented in the current study. Specifically, dual unsupervised *k*-means clustering, a cross-shaped verification, an intersection operation, a connected component analysis and a patch expansion are successively executed. It is well known that most of the supervised machine learning (e.g., SVM, random forest, CNN) methods require a huge amount of labeled or annotated data usually produced by experts manually. For example, 12,481 lesion patches and 16,741 normal patches are derived from the manually segmented regions in the study by Yao et al. [28]. In the experiments conducted by Song et al. [31], a total of 2062 2-D annotated ROIs were manually drawn by two radiologists. This manual annotation process is always time-consuming and high-costing. In this study we have proposed a semi-supervised method for generating annotated image patches which could effectively and efficiently help radiologists and researchers get rid of the tedious manual annotations. However, the comparison of this method with the manual annotations conducted by the medical experts remains unexplored.

### A simplified or deep CNN, and parameter optimization

We have proposed and implemented a simplified CNN model consisting of only a convolutional layer, a pooling layer and two fully connected layers with a Softmax layer. Comparing with LeNet [36] of two convolutional layers, AlexNet [37] of eight learned layers, and VGG-VD [38] of 19 layers, our CNN model is very “shallow”. However, its sensitivity of 98.9% makes the exploration of more deep CNN models not so urgent. The high sensitivity clearly justifies the ability of the proposed framework to segment lung parenchyma without many difficulties. For the more complicated lesions, the deep CNN is required because the network with more depth can better approximate the target function with high nonlinearity and achieve better feature representations [39]. For instance, Anthimopoulos et al. had proposed one deep CNN with five convolutional layers to do lung pattern classification for interstitial lung diseases [32]. Recently Shin et al. [40] have explored three CNN architectures of CifarNet, AlexNet and GoogLeNet using lymph node (LN) detection and interstitial lung disease (ILD) classification.

As done in some previous CNN studies [32, 40], the optimization of the CNN hyper-parameters is critical and inevitable. Most trends of influence of hyper-parameters on the training accuracy observed in the current study accord with previous study. More specifically, as the common choice for most CNNs, the maximum pooling yields high accuracy and is much faster in terms of convergence. It is found that the dropout and normalization are effective to accelerate the convergence. Small kernel size of 5 × 5 is better than 10 × 10. Even smaller kernels have been employed, e.g., 3 × 3 kernel in VGG-net, 2 × 3 kernel in work by Anthimopoulos et al. [32]. A relatively larger number of convolutional kernels and output units of FC, and a relatively smaller batch size and number of epochs lead to high accuracy. It is of great importance to mention that the learning rate is a very important parameter whose value needs to assign with special attention and according to the size of the objects of interest contained in the image patches to be classified.

### The proposed method comparison with the traditional methods

To the end of detecting and analyzing the various lung diseases, numerous lung segmentation methods have been proposed. Given the wide range of lung lesions, the existing methods aimed to solve different problems and they have been implemented on different image types acquired from various databases. Thus, it is quite challenging to reproduce these algorithms as well as to collect the dataset used in their experiments.

Aiming to quantitatively evaluate the performance of our proposed method, extensive experiments have been conducted using the proposed method and four commonly used lung segmentation methods including the iteration, improved Ostu, watershed and region growth methods [12]. The segmentation results achieved by each of the five methods were evaluated considering the Dice similarity coefficient (*DSC*) and the algorithm self-adaptability measure. The algorithm self-adaptability can be defined as the number of images successfully segmented by the system after inputting a set of images. The computed *DSC* and self-adaptability values have been recorded in Table 4. A comparison of those values shows that our method achieved a *DSC* of 0.9671 which is greater than that of the other four methods. In addition, our fully automatic machine learning based method yielded an adaptability of 100% because of the contribution of the CNN model while the iteration, improved Ostu, watershed and region growth methods yielded an adaptability of 83.33%, 83.68%, 62.5% and 91.32%, respectively. The iteration and improved Ostu methods failed to segment all the input images because of the presence of images whose cylinder-shaped background CT value and injected contrast agent value is always lower than − 1024HU and higher than 1024HU, respectively. The watershed method could not segment all the images due to the fact that the boundary of lung parenchyma is not stable and is easier to leak into the human body. Therefore, the proposed method is superior to the state-of-the-art lung segmentation methods both effectively and efficiently.

**Table 4:** Comparison of the proposed method with the traditional methods

Methods	DSC (vs observer A) (%)	DSC (vs observer B)	Average DSC	Self-adaptability
Iteration	95.40	95.22	95.31	83.33
Improved Ostu	95.35	95.18	95.27	83.68
Watershed	94.72	94.49	94.61	62.50
Region growing	96.65	96.50	96.58	91.32
Proposed method	96.80	96.62	96.71	100

### Parenchyma at first, then the whole lung analysis in a unified framework

To the best of our knowledge, this is the first study conducted on extracting lung parenchyma from CT images using a fully machine learning-based framework, rather than the whole lung or various lung pathologies. This idea originated from one previously ignored fact that lung parenchyma is quite different from lung pathologies [11, 12, 41]. The lung parenchyma owns commonalities across subjects, diseases and CT scanners although lung pathologies exist under various appearances. An accurate segmentation of lung parenchyma may have potential to help locate and analyze the lung lesions. Current framework is compatible to further segmentation of various lesions, so the whole lung analysis might be done in a unified framework.

Our method naturally belongs to the bottom-up strategy in which only local information of the shape, texture and intensity within a 32 × 32 patch is considered. The ROI is larger than the thresholding and region-based approaches. Multiple scale technique (e.g., using the patches in different sizes simultaneously) may further improve the performance. Comparing to the top-down strategy (the model-based and neighboring anatomy-guided methods), our method presents lower computational time and can successfully segment lung with a certain level of abnormities. Last, not the least, the CNN-based methods get rid of the work of feature engineering. All the features for classification are learned from the training data and no handcrafted feature is necessary [42].

### Pixel-wise or patch-wise segmentation

**Fig. 8:**
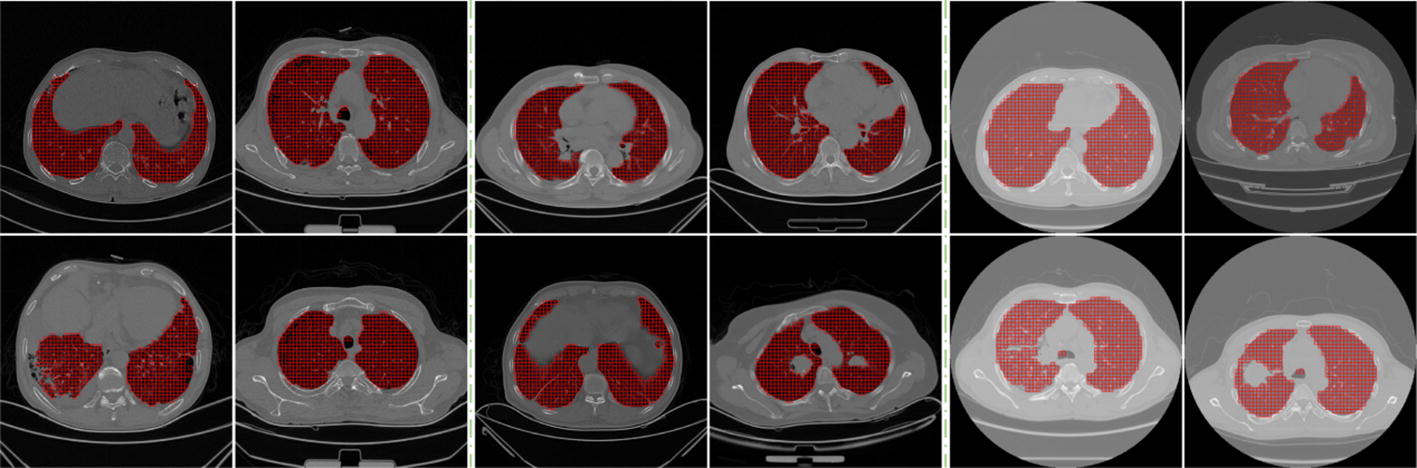
Segmentation of lung parenchyma using small patches

Using the proposed CNN model, the average lung parenchyma regions segmentation time is estimated at 10.75 s for each slice with 512 × 512 voxels, which is a pixel-wise segmentation. The computational time can be shortened by using a multiple-core or GPU provided computer. Another alternative might be the patch-wise segmentation. For instance, one can split the CT images into 8 × 8 patches, generate 32 × 32 patches, and input them into the trained CNN to realize the segmentation of lung parenchyma, whose average computational time can be shorten to 0.16 s for each slice. Figure 8 gives some relevant examples. The lung parenchyma regions can also be segmented with reasonable accuracy for subjects with COPD (the first and second columns) and lung cancer (the third to sixth columns). The images in the third and fourth columns of Fig. 8 are obtained using CT scanners, and the others in the fifth and sixth columns are obtained from PET/CT scanners. The boundaries between lung parenchyma and lung tumor, pleural effusion, pulmonary bulla and other backgrounds can be depicted well. Therefore, our proposed segmentation framework owns the good feature of suitability of the multiple-resolution strategy.

Although our proposed segmentation method achieved quite satisfactory performance, it presents some limitations that are worth mentioning. First, the size of the patches utilized in our CNN model is fixed as 32 × 32. The effect of the patch size on the CNN performance is not investigated. Moreover, ensemble of CNN models fed with patches of different sizes may help integrate multilevel features [43]. Second, some state-of-art CNN models such as Fast RCNN and Mask RCNN have presented excellent performance for the objection detection and segmentation [44, 45]. However, their performances on the dataset generated by our clustering based method remain unexplored. In other words, these CNN models may do well in the lung parenchyma segmentation. Third, though we had built up one fully machine learning-based framework, some instances including segmentations of pulmonary nodules, consolidation, and pleural effusion need to be developed. Combination of segmentations of lung parenchyma and various lesions will demonstrate the power of our fully machine learning-based framework.

## Conclusions

A novel machine learning-based method has been presented to segment lung parenchyma from CT images automatically. To the best of our knowledge, it is the first study conducted on extracting lung parenchyma from CT images, rather than the whole lung or various lung pathologies using a fully machine learning-based framework. Moreover, a clustering method is used to automatically generate huge amount of annotated data. This clustering algorithm can properly and efficiently replace the tedious manual annotations which could significantly reduce the workload of the radiologists leading to a more accurate and faster diagnosis of the diseases. The CNN parameters have been carefully optimized through extensive experiments. Through the trained CNN, the voxel-wise identification of lung parenchyma can be achieved without any feature engineering work. Besides the cross-validation, an independent dataset of more than 200 subjects with lung cancer or COPD, acquired by CT or PET/CT scanners have been used to evaluate the performances of the CNN model. The quantitative results show that our method can segment lung parenchyma from images acquired through different imaging modalities (i.e., CT and PET/CT) with very satisfactory performance. The proposed machine learning-based framework may have the potential to help locate and analyze the lung lesions.
